# Artificial intelligence significantly improves the diagnostic accuracy of deep myxoid soft tissue lesions in histology

**DOI:** 10.1038/s41598-022-11009-x

**Published:** 2022-04-28

**Authors:** Maximus C. F. Yeung, Ivy S. Y. Cheng

**Affiliations:** 1 Department of Pathology, The University of Hong Kong, Queen Mary Hospital, 11/F, Block T, 102 Pokfulam Road, HKSAR, Hong Kong; 2 Department of Pathology, CUHK Medical Centre, 9 Chak Cheung Street, Shatin, New Territories, HKSAR Hong Kong

**Keywords:** Cancer, Sarcoma

## Abstract

Deep myxoid soft tissue lesions have posed a diagnostic challenge for pathologists due to significant histological overlap and regional heterogeneity, especially when dealing with small biopsies which have profoundly low accuracy. However, accurate diagnosis is important owing to difference in biological behaviors and response to adjuvant therapy, that will guide the extent of surgery and the need for neo-adjuvant therapy. Herein, we trained two convolutional neural network models based on a total of 149,130 images representing diagnoses of extra skeletal myxoid chondrosarcoma, intramuscular myxoma, low-grade fibromyxoid sarcoma, myxofibrosarcoma and myxoid liposarcoma. Both AI models outperformed all the pathologists, with a significant improvement of accuracy up to 97% compared to average pathologists of 69.7% (p < 0.00001), corresponding to 90% reduction in error rate. The area under curve of the best AI model was on average 0.9976. It could assist pathologists in clinical practice for accurate diagnosis of deep soft tissue myxoid lesions, and guide clinicians for precise and optimal treatment for patients.

## Introduction

Myxoid lesions are a group of heterogeneous soft tissue tumour that are characterized by presence of marked extracellular mucoid matrix in the stroma^1^. Various deep myxoid soft tissue tumours exhibit different biological behaviour, including benign, locally aggressive with tendency for local recurrence, and malignant ones that may metastasize. The diagnosis usually requires image guided or open biopsy to guide subsequent treatment, such as the need for neo-adjuvant therapy or the extent of surgical treatment. Relatively common entities occurring in deep soft tissue requiring biopsy for diagnosis include intramuscular myxoma, myxofibrosarcoma, myxoid liposarcoma, low-grade fibromyxoid sarcoma and extraskeletal myxoid chondrosarcoma.

Intramuscular myxoma is a benign hypocellular soft tissue tumour composed of bland spindle-shaped cells embedded in a usually abundant myxoid and hypovascular stroma. Activating point mutation in *GNAS* gene is detected in > 90% of sporadic intramuscular and cellular myxomas as well as in cases of Mazabraud syndrome^2^. Myxofibrosarcoma has multinodular architecture with infiltrative margins and contains variably prominent pleomorphic cells in a myxoid stroma with distinctive curvilinear vessels^3^. Karyotypes are highly complex, with intratumoural heterogeneity and chromosome numbers in the triploid or tetraploid range in most cases. Local, often repeated recurrences, unrelated to histological grade, occur in 30–40% of cases, usually as a result of inadequate surgery, sometimes even in experienced hands. Myxoid liposarcoma (MLS) is a malignant tumour composed of uniform, round to ovoid cells with variable numbers of small lipoblasts, set in a myxoid stroma with a branching capillary vasculature. Translocations producing *FUS::DDIT3* or rarely *EWSR1::DDIT3* fusion transcripts are pathognomonic^4^. Round cell liposarcoma is a high-grade variant of this tumour, with increased propensity for metastasis to other soft tissue sites and bone^5^. MLS is extremely radiosensitive^6^ and relatively sensitive to anthracyclines and trabectedin compared with other sarcomas. Therefore, neo-adjuvant chemotherapy and radiotherapy can be beneficial in treating this tumour^7^. Low-grade fibromyxoid sarcoma is a malignant fibroblastic neoplasm characterized by alternating collagenous and myxoid areas, deceptively bland spindle cells with a whorling growth pattern, and arcades of small blood vessels. These tumours consistently have either *FUS::CREB3L2*^8^ or *FUS::CREB3L1*^9^ gene fusions. Diffuse strong MUC4 expression is noted in nearly all cases^10^. Extraskeletal myxoid chondrosarcoma (EMC) is a malignant mesenchymal neoplasm of uncertain differentiation characterized by abundant myxoid matrix, multilobular architecture, and uniform cells arranged in cords, clusters, and reticular networks. These tumours are characterized by *NR4A3* gene rearrangement^11^. Despite the name, there is no evidence of cartilaginous differentiation.

It may not be difficult to distinguish these entities with excision specimens by a general pathologist. However, with regional variations in morphology, focal areas of each entity may resemble others. This is especially the cases with small biopsies where only tiny bits of tumour are sampled. While advances in molecular pathology allow pathologists to have more accurate diagnosis with the ancillary tests e.g. MUC4 stain for low-grade fibromyxoid sarcoma and *FUS::DDIT3* fusion transcript for myxoid liposarcoma, not all hospitals have access to these molecular tests, and sometimes the tissue samples in small biopsies may be too scanty for further ancillary tests, so the diagnoses are mainly based on the histomorphology. On the other hand, diagnosis of myxoid lesions without ancillary tests, especially in small biopsy, are prone to significant interobserver and intraobserver variability, leading to overall low accuracy^12^. Differentiating between low grade myxofibrosarcoma and intramuscular myxoma is particularly problematic, as there are markedly hypocellular areas in the low grade myxofibrosarcoma that are virtually indistinguishable from intramuscular myxoma by human eye in small biopsies. The nuclear pleomorphism that is the main discriminator between the two entities may not be sampled, and it is a subjective assessment which has high interobserver variability. Therefore, patients with low grade myxofibrosarcoma may be under-diagnosed as benign intramuscular myxoma, leading to inadequate margins with multiple recurrences, and definite features of low grade myxofibrosarcoma can only be found in subsequent excision. And cellular myxoma may be misdiagnosed as myxofibrosarcoma, leading to over-treatment and unnecessary adjuvant therapy in some cases.

Accurate diagnosis of these deep myxoid soft tissue lesions in small biopsies is important, as they guide the subsequent surgical plan and management. For example, if it is an intramuscular myxoma, the patients may opt for observation^13^ or marginal excision^14^, while myxofibrosarcoma needs a wider surgical margin to avoid recurrence^15^. On the other hand, since myxoid liposarcoma is extremely radiosensitive, neoadjuvant radiotherapy may be given to the patients^16^.

Artificial intelligence, specifically deep convolutional neural networks (CNNs), has been increasingly applied in the field of computational pathology^17^ to assist diagnosis^18^, classification^19^ and predicting outcomes^20^. It involves multiple convolutional processing layers that can learn numerous levels of abstraction for data representation. Multiple filters were applied to extract different local features. The resultant accuracy with current state-of-the-art models could in some cases, even exceed human performance. It can provide more consistent analysis of images to reduce the interobserver and intraobserver variability in diagnosis. In this study, we try to train two CNNs to diagnose deep myxoid soft tissue lesions and compare their performances with human pathologists.

## Results

### Pathologists performance

**Figure 1:**
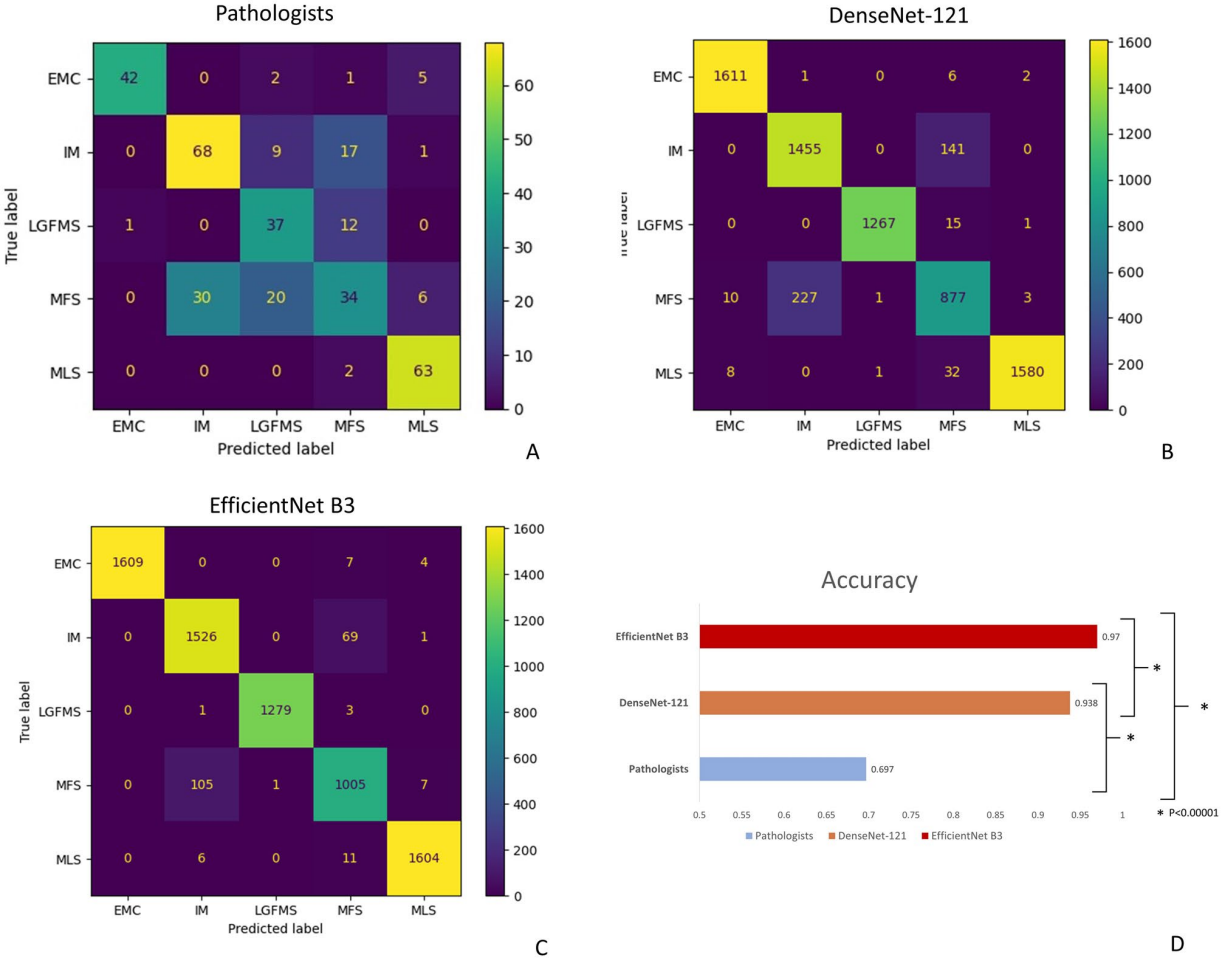
Confusion matrix of pathologists (**A**), DenseNet-121 (**B**) and EfficientNet B3 (**C**). Both deep learning models have significant improvement in accuracy compared to pathologists (**D**), and EfficientNet B3 has a small significant further improvement compared to DenseNet-121. *EMC* extraskeletal myxoid chondrosarcoma, *IM* intramuscular myxoma, *LGFMS* low grade fibromyxoid sarcoma, *MFS* myxofibrosarcoma, *MLS* myxoid liposarcoma.

A total of 350 H&E stained slides containing a mixture of cases from biopsies and excisional specimens with diagnosis of intramuscular myxoma (IM), myxofibrosarcoma (MFS), myxoid liposarcoma (MLS), low-grade fibromyxoid sarcoma (LGFMS) and extraskeletal myxoid chondrosarcoma (EMC), were evaluated by five pathologists with different years of post-fellowship experiences. Basic information, like the age, gender and site of lesion, was given. The pathologists read the whole glass slides and gave the favoured diagnoses from one of the above 5 entities. The overall accuracy of pathologists in diagnosing these deep myxoid lesion was only 69.7%, which was worse in biopsy samples for each category, with overall accuracy of only 63.2% (Figs. 1A, 3A). The performance was especially poor in differentiating between intramuscular myxoma, myxofibrosarcoma and low-grade fibromyxoid sarcoma, with a recall rate of myxofibrosarcoma being only 0.38. On the other hand, diagnosing extraskeletal myxoid chondrosarcoma and myxoid liposarcoma, which had a more distinct histological features, had a generally better performances for the pathologists.

### AI model performance

**Figure 2:**
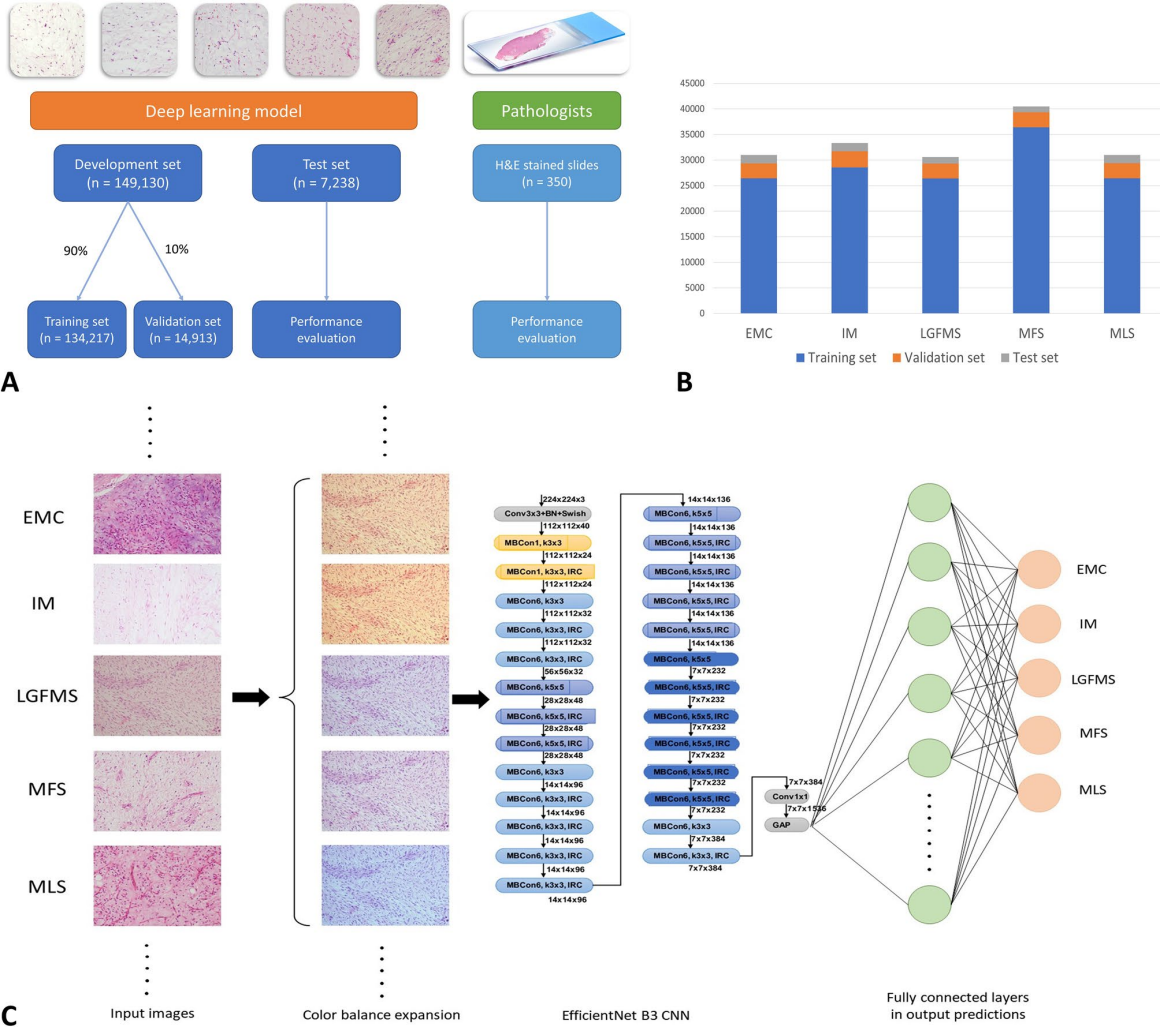
Overview of strategies of training the deep learning models in comparison with pathologists (**A**). Number of images in different diagnostic categories of different datasets (**B**). Schematic diagram depicting the training workflow of AI model (**C**). *EMC* extraskeletal myxoid chondrosarcoma, *IM* intramuscular myxoma, *LGFMS* low grade fibromyxoid sarcoma, *MFS* myxofibrosarcoma, *MLS* myxoid liposarcoma.

The development set of 149,130 images was taken from cases of the 5 diagnoses from Queen Mary Hospital, a tertiary referral center and university teaching hospital. They were divided into training set (90%) and validation set (10%). An independent test set of 7238 images taken from different cases from a peripheral hospital was used to evaluate the generalizability and final performance of the model (Fig. 2A,B). Two deep learning models were developed, which were based on convolutional neural network (CNN) of EfficientNet B3 and DenseNet-121, respectively, with the top layers both replaced by a customized fully connected network with 5 prediction outputs corresponding to the 5 diagnoses (See “Methods”; Fig. 2C). The models achieved the highest validation accuracy of 0.9961 and 0.9943, respectively. These models were used as final model performance evaluation on an independent test set from the peripheral hospital.

**Figure 3:**
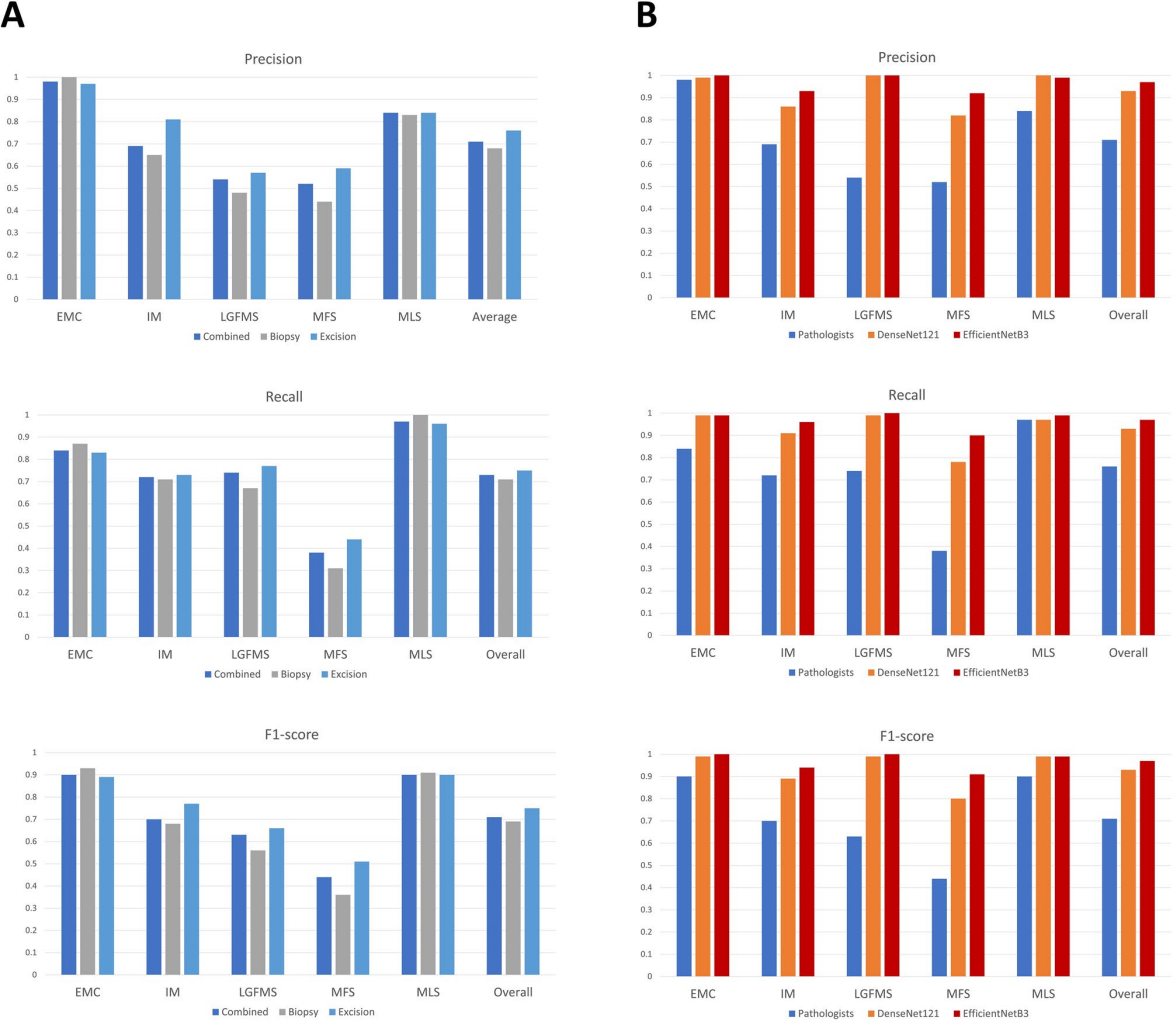
The precision, recall and F1-score for different diagnostic categories of pathologists (**A**) and the deep learning models (**B**). *EMC* extraskeletal myxoid chondrosarcoma, *IM* intramuscular myxoma, *LGFMS* low grade fibromyxoid sarcoma, *MFS* myxofibrosarcoma, *MLS* myxoid liposarcoma.

**Figure 4:**
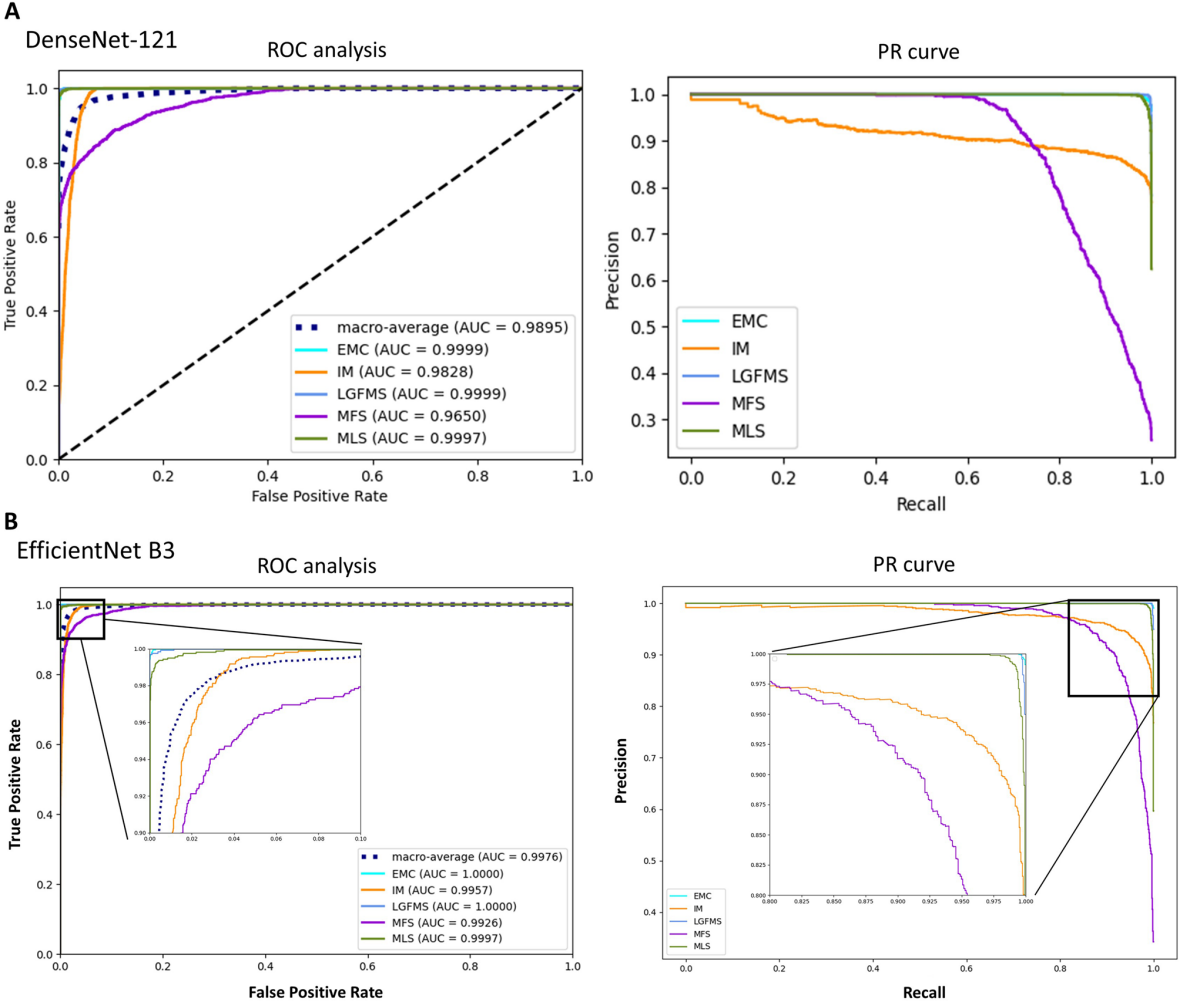
Receiver operating characteristic^25^ curve and Precision-Recall^28^ curve of DenseNet-121 (**A**) and EfficientNet B3 (**B**). *EMC* extraskeletal myxoid chondrosarcoma, *IM* intramuscular myxoma, *LGFMS* low grade fibromyxoid sarcoma, *MFS* myxofibrosarcoma, *MLS* myxoid liposarcoma.

As seen in the confusion matrix and performance indices (Figs. 1B–D and 3B), the overall accuracies of both EfficientNet B3 and DenseNet-121 deep learning models had been significantly improved to 0.97 and 0.938, respectively on the test set, compared to 0.697 of pathologists (p < 0.00001), corresponding to 90% reduction in error rate. In particulars, the performance in differentiation between IM, MFS and LGFMS had been markedly enhanced, with recall rate of more than 0.9 in EfficientNet B3 for these entities, while that of EMC and MLS were almost perfect with a recall rate of 0.99. The Area under the Receiver Operating Curve (AUROC) for all classes combined was astonishingly achieved to be 0.9976 and 0.9895, respectively (Fig. 4). A small but significant improvement of performance for EfficientNet B3 was seen compared to DenseNet121. There was still some mix-up for a tiny fraction of images in differentiation between IM and MFS, but these represented some extreme close-up regions where they closely mimic each other.

## Discussion

Deep myxoid soft tissue lesions are a group of tumour characterized by abundant myxoid substances in the stroma. Relatively common entities include intramuscular myxoma, myxofibrosarcoma, myxoid liposarcoma, low-grade fibromyxoid sarcoma and extraskeletal myxoid chondrosarcoma. While a competent pathologist should have little difficulty in distinguishing these entities on excisional specimens, it may be challenging on small biopsies, due to morphological heterogeneity both within the tumours and between tumours of same entities. In practice, ancillary tests could be performed to assist diagnosis. For example, MUC4 staining can be done to exclude low-grade fibromyxoid sarcoma. With advancement in molecular understanding of the sarcoma, more specific translocations are discovered in different sarcomas. For instance, myxoid liposarcoma is characterized by *DDIT3* translocation, most commonly fused with *FUS* gene, while extraskeletal myxoid chondrosarcoma features a recurrent *NR4A3* translocation, most frequently translocated to *EWSR1* gene. While these molecular signatures are specific enough for definite diagnosis, not all medical laboratories have access to these molecular tests, and sometimes the tissue in small biopsies may be too scanty or too severely degraded that make it unsatisfactory for the molecular tests.

In our test slides for pathologists, they have a relatively good performance in diagnosing extraskeletal myxoid chondrosarcoma and myxoid liposarcoma. This is not surprising, as their morphologies are relatively distinctive among the five diagnoses, with the former having cords of more epithelioid cells, while the latter having characteristic chicken-wire vascular network. On the other hand, distinguishing between intramuscular myxoma, low-grade fibromyxoid sarcoma and myxofibrosarcoma is shown to be quite troublesome for the pathologists. In particular, the recall rate for myxofibrosarcoma is particularly low (0.38), as the hypocellular area sampled in small biopsy has been mis-diagnosed as intramuscular myxoma. The nuclear pleomorphism, that is characteristic in myxofibrosarcoma, is a subjective feature and may not be well-sampled in small biopsy, and most pathologists are not willing to make the diagnosis unless the pleomorphism is convincing. On top of that, the pathologists have tendency to over-diagnose low-grade fibromyxoid sarcoma with morphology alone, when the MUC4 results are not available and their unfamiliarity of this entity.

**Figure 5:**
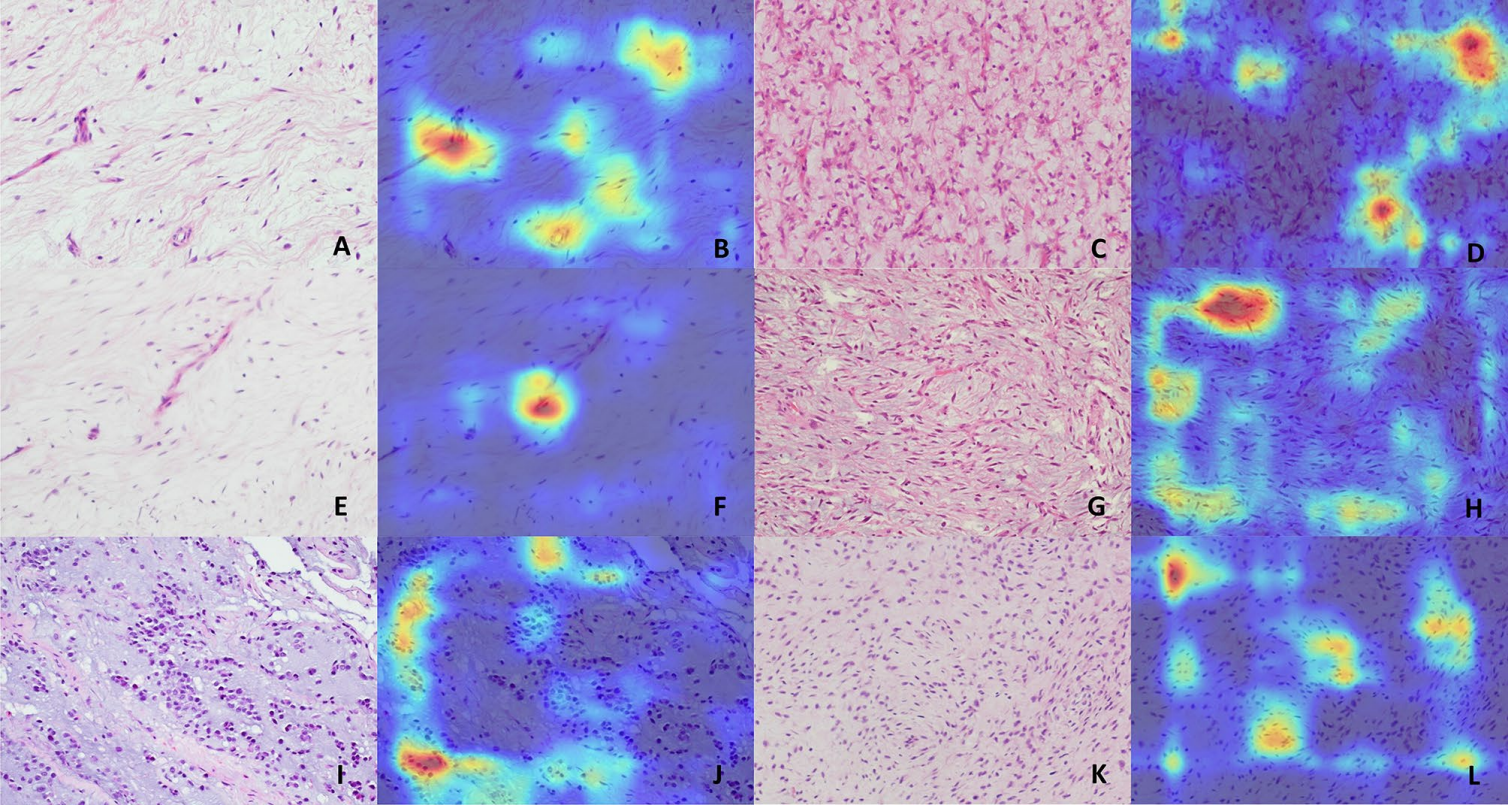
Example images and heatmaps generated by the deep learning model of (**A**,**B**) Intramuscular myxoma, (**C**,**D**) Myxoid liposarcoma, (**E**–**H**) Myxofibrosarcoma, (**I**,**J**) Extraskeletal myxoid chondrosarcoma, and (**K**,**L**) Low-grade fibromyxoid sarcoma. The images are mainly activated over tumour nuclei and the vessels. There are subtle sub-visual differences of tumour nuclei and vessels, especially between intramuscular myxoma and myxofibrosarcoma, that are not noticeable by human eyes.

On the other hand, artificial intelligence (AI) using deep learning model with convolutional neural network have been demonstrated to outperform pathologists in diagnosing deep myxoid lesions. This illustrates the potential of AI to augment the pathologist’s eye with information or intelligence that cannot be done by human examination^21^. We choose to train the models with images taken at 20X objectives (200X magnification), as the primary aim of the model is to implement it in small biopsies, in which usually only small amount of tumour area was present. Without low power architecture interpretation as done in routine histological analysis by pathologists, the AI system clearly excels by just looking at small area of tumour at relatively higher power. This may be explained by the ability of AI to mine ‘sub-visual’ image features^22^, such as tumour cell-stroma interaction, that may not be visually discernible by a pathologist. From the activation heatmaps of different diagnostic images (Fig. 5), vessels patterns and nuclear morphology appear to be the main contributing factors for diagnosis, which is congruent with what the pathologists will assess. However, computer vision is able to gather more sub-visual information with diagnostic relevance from an image section than a pathologist, offering better diagnostic power. In particular, the performance boost is especially prominent for myxofibrosarcoma, increasing the recall rate from 0.38 to 0.9. There may be subtle differences between the bland looking region of low grade myxofibrosarcoma and true intramuscular myxoma that is beyond the comprehension of pathologists. The cytomorphology that is deemed to be myxofibrosarcoma by the AI models will also be more consistent, instead of mere subjective nuclear pleomorphism determined by the human eyes. This will provide more congruous diagnosis to avoid over- or under-diagnosis. Nevertheless, there remains small number of cases, especially between intramuscular myxoma and low grade myxofibrosarcoma, where these two entities are truly undifferentiable, even by AI.

Both deep learning models developed in this study have outperformed pathologists, but EfficientNet B3 further boosts a significant improvement in accuracy to 97% compared to DenseNet-121 of 93.8%. DenseNet CNNs was developed in 2017^23^ and composed of multiple dense blocks in which each layer obtains additional inputs from all preceding layers and passes on its own feature-maps to all subsequent layers. Compared to the traditional CNNs, this allowed a strong gradient flow of error signal, fewer parameter requirement with lower complexity features and higher computational efficiency. EfficientNet^24^ was one of the most potent CNN architectures recently developed by Google, which utilized a compound scaling method to enlarge the network depth, width, and resolution. It encompassed models of different complexities to suit different classification tasks, from B0 to B7, and had obtained state-of-the-art capacity in various benchmark datasets while requiring fewer computation resources compared to other models. In the classification of 1000 categories in ImageNet dataset, these two models achieved a top-1 accuracy of 74.9% and 81.6%, respectively. The advance in performances of EfficientNet in natural images of ImageNet dataset was also exemplified in histological images with transfer learning, so the fundamental improvement in algorithmic architectures was shared in different image types. Newer CNNs are continuously developed, such as Normalizer Free Nets (NFNETS) published last year in 2021^25^, and they continuously break record in the ImageNet top-1 accuracy performance up to 86.5%. We are looking forward to seeing this improvement will also be beneficial to digital pathology.

### Limitations

Currently, the deep learning models had only been trained to classify one of the five relatively common deep myxoid soft tissue tumours – intramuscular myxoma, myxofibrosarcoma, myxoid liposarcoma, low-grade fibromyxoid sarcoma and extraskeletal myxoid chondrosarcoma. Tumours other than the five included in the model would definitely not be able to be predicted correctly, for examples, some myxoid variants of entities like myxoid dermatofibrosarcoma protuberens, myxoid leiomyosarcoma and myxoid nerve sheath tumour, as well as some extremely rare tumours can also have myxoid stroma as predominant features e.g. myxoinflammatory fibroblastic sarcoma and ossifying fibromyxoid tumour. However, routine immunohistochemical stainings available in nearly all clinical laboratories could easily exclude some of the myxoid variants entities, like S100 to exclude myxoid nerve sheath tumour. Also, those extremely rare entities had some peculiar features for specific diagnosis. For instance, ossifying fibromyxoid tumour had a complete or incomplete bone shell at the periphery, and myxoinflammatory fibroblastic sarcoma had some bizarre virocyte-like cells and pseudolipoblast in an inflamed background.

Since the model was only trained with images taken with 20X objective, images taken at other magnification might not be correctly classified. Also, it should be emphasized that neural network model was only as good as the training data. Due to heterogeneity of tumour, our datasets might not encompass all the variations encountered in these tumour entities.

## Methods

### Preparation of images datasets

Images were taken from slides of a mixture of cases from Queen Mary hospital (QMH) and United Christian Hospital (UCH), with diagnosis of intramuscular myxoma, myxofibrosarcoma (low to intermediate grade), myxoid liposarcoma (including high grade round cell component), low-grade fibromyxoid sarcoma and extraskeletal myxoid chondrosarcoma, by a camera (Nikon model DS-FI3) attached to the microscope. To ensure there was a balance of tumour architecture and cytology details, and to suit our ultimate use for small biopsy cases, they were taken with eyepiece of 10X and objective of 20X magnification. Intramuscular myxoma, myxoid liposarcoma and extraskeletal myxoid chondrosarcoma were molecularly confirmed with presence of *GNAS* mutation by Sanger’s sequencing, presence of *FUS::DDIT3* transcripts by RT-PCR and presence of *NR4A3* translocation by FISH breakapart probes, respectively. Cases of low grade fibromyxoid sarcoma had shown diffuse strong staining for MUC4. For cases of myxofibrosarcoma, apart from the compatible morphology, they were demonstrated to be negative for *GNAS* hotpot mutation and MUC4 staining. In order to minimize the effects of data imbalance, more images were taken from less common entities e.g. LGFMS and EMC, and fewer images were taken from more common entities e.g. MFS. The development set of images from QMH were divided into training set (90%) and validation set (10%). An independent test set from UCH was used to evaluate the final performance of the model.

### Images preprocessing

In order to eliminate the potential effects of white balance in affecting the interpretation, five images of different color balance were generated from each original image^26^. This produced more realistic color shift from incorrect white balance settings from onboard cameras or color source of microscope, compared to existing color augmentation methods. As a result, each tumour histology would have 6 images of different color balance for training. Image augmentation was implemented using random horizontal and vertical flipping, random rotation, random zoom, random translation and random change in contrast. The images were resized to 300 × 300 px for EfficientNetB3 and 224 × 224 px for DenseNet-121, and rescaled to a floating value range of [0, 1.0].

### Deep learning model

Our models were implemented using EfficientNet B3 and DenseNet121 as the base deep learning model architecture, with the original top layers of both CNNs being discarded. The output layers were processed with a custom Global Average Pooling layer followed by a Dropout layer, a fully connected layer with 128 nodes using Relu activation, and finally a fully connected layer of 5 output nodes corresponding to one of the prediction classes, using softmax activation. Transfer learning was initially performed using pretrained ‘noisy student’ weights on ImageNet. The base layers were initially frozen and trained with Adam optimizer, using an initial learning rate of 0.0001, for 10 times. Subsequently, the whole model, except for the Batch Normalization layers, were unfrozen and further trained with Adam optimizer using initial learning rate of 0.00004. A batch size of 64 was used. The learning rate would be reduced by half if the validation loss was not lowered after 4 epochs. Training would be stopped earlier when the validation loss did not change after 12 epochs. The model with the lowest validation loss was utilized as the final model.

### Pathologists testing

A total of 350 H&E stained slides containing a mixture of cases from biopsies and excisional specimens with diagnosis of intramuscular myxoma, myxofibrosarcoma, myxoid liposarcoma, low grade fibromyxoid sarcoma and extraskeletal myxoid chondrosarcoma, were evaluated by five pathologists with different years of post-fellowship experiences. Basic information, like the age, gender and site of lesion, was given. The pathologists read the whole glass slides and gave the favoured diagnoses from one of the above 5 entities.

### Statistical analysis

The model was implemented and trained using TensorFlow^27^. Precisions, recalls, F1-score, accuracy, confusion matrix, and AUCs were calculated in python using the scikit-learn package^28^. The graphs were plotted using matplotlib^29^. Statistical significance of difference in accuracy performance was calculated by Chi-square test.

## Data Availability

The images datasets used and/or analyzed during the current study are available from the corresponding author on reasonable request. The Python scripts for training the model, as well as the trained model with its weights are uploaded to the github repository at http://www.github.com/maximus3219.
